# Blood biomarkers for Alzheimer’s disease: Key challenges of clinical implementation

**DOI:** 10.4103/NRR.NRR-D-25-00569

**Published:** 2025-09-29

**Authors:** Rafaela Luiza C. Franco, Tai R. Hunter, Fernanda G. De Felice

**Affiliations:** D’Or Institute for Research and Education, Rio de Janeiro, Brazil; Institute of Medical Biochemistry Leopoldo de Meis, Federal University of Rio de Janeiro, Rio de Janeiro, Brazil; Department of Biomedical and Molecular Sciences, Queen’s University, Kingston, ON, Canada; Center for Neuroscience Studies and Department of Psychiatry, Queen’s University, Kingston, ON, Canada

Alzheimer’s disease (AD) is a complex, progressive neurodegenerative disorder and the leading cause of dementia worldwide. It is characterized by the accumulation of extracellular amyloid–beta (Aβ) plaques and intracellular tau neurofibrillary tangles, leading to synaptic dysfunction, neuronal loss, and cognitive decline. These pathological changes can begin decades before clinical symptoms emerge, highlighting the critical need for early, accessible, and accurate diagnostic tools. Traditionally, AD diagnosis has relied on clinical assessments supported by neuroimaging and cerebrospinal fluid (CSF) biomarkers, which are accurate but costly and invasive (Jack et al., 2024). However, recent advancements in blood-based biomarkers (BBMs) have opened a new frontier in AD diagnostics, offering a minimally invasive, cost-effective, and scalable approach to early detection and disease monitoring (Hunter et al., 2025). The 2024 Alzheimer’s Association guidelines mark a turning point by recognizing BBMs as core diagnostic tools alongside amyloid positron emission tomography (PET) imaging and CSF biomarkers (Jack et al., 2024). Here, we discuss the potential of BBMs in AD diagnosis, the technological advancements driving their development, and the key challenges that remain for their widespread clinical implementation.

**Neuroimaging and CSF biomarkers in AD:** Neuroimaging and CSF biomarkers are key approaches in AD diagnosis. Magnetic resonance imaging reveals patterns of brain atrophy, while PET with amyloid or tau tracers visualizes pathological changes. CSF analysis measures Aβ and tau proteins, reflecting more specific biochemical changes in AD. These methods support early and accurate diagnosis but have limitations that restrict broader clinical use. Structural magnetic resonance imaging primarily detects atrophy, a condition common to various neurodegenerative diseases besides AD, such as Lewy body dementia and vascular dementia. As such, its use is insufficient for diagnosing mild cognitive impairment or dementia due to AD, although it remains useful for general clinical workups and monitoring responses to anti-amyloid immunotherapy. While the development of amyloid and tau PET tracers has improved diagnostic specificity for AD, PET imaging remains expensive, is not routinely used in clinical settings, and amyloid PET cannot distinguish AD from other amyloid-positive conditions (Hunter et al., 2025). CSF biomarkers provide valuable insights into AD pathology because of their direct interaction with the brain. However, the lumbar puncture used to collect CSF is met with patient and clinician reluctance due to its invasiveness and associated risks, including headaches, back pain, and in rare cases, infections or neurological complications. Additionally, CSF acquisition is not a routine clinical procedure, as it is limited to specialized clinics, and obtaining ethical approval for research or clinical trials can be challenging. These limitations have driven interest in less invasive, more accessible, and cost-effective diagnostic alternatives, with BBMs as a promising approach.

**Challenges and advancements in blood-based detection of central nervous system biomarkers:** Detecting central nervous system (CNS)–derived biomarkers in blood is challenging due to the blood–brain barrier and the dilution effect caused by the high blood-to-CSF volume ratio. These factors significantly reduce the concentration of CNS biomarkers in the bloodstream, making their detection difficult using conventional methods. However, recent advancements in ultrasensitive immunoassay technologies, such as the Single Molecule Array (SiMoA®), have greatly improved the ability to detect CNS-derived proteins in blood. SiMoA is an automated, bead-based enzyme-linked immunosorbent assay platform that uses femtoliter wells to capture individual beads, enabling detection at the single-molecule level, thereby increasing sensitivity. This technology enables the measurement of AD-related biomarkers, including Aβ_42_, Aβ_40_, and phosphorylated tau (p-tau) species, at sub-femtomolar concentrations, improving diagnostic accuracy. Other technologies, including chemiluminescence-based assays, electrochemiluminescence platforms, and mass spectrometry-based techniques, have also shown significant improvements in detecting CNS biomarkers with high precision (Hunter et al., 2025).

Understanding the comparative strengths of these platforms is critical for their successful clinical adoption. SiMoA offers high sensitivity, automation, and compatibility with small sample volumes, making it suitable for clinical settings. In contrast, immunoprecipitation coupled with mass spectrometry has shown superior specificity for quantifying plasma Aβ isoforms but remains labor-intensive and less suited to high-throughput workflows (Janelidze et al., 2021). These cutting-edge technologies represent a significant advancement in the development of blood-based diagnostics for AD, though the optimal choice depends on clinical goals and resources.

**Figure 1:**
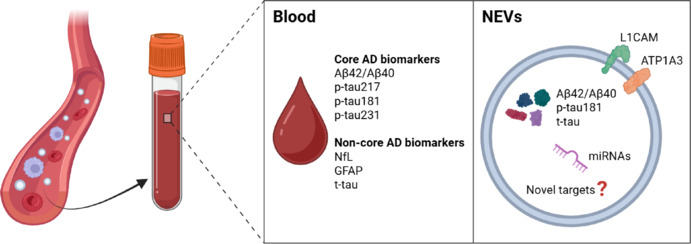
Blood-based and NEVs biomarkers in AD. Schematic representation of core (Aβ42/Aβ40, p-tau217, p-tau181, and p-tau231) and non-core (NfL, GFAP, and t-tau) AD biomarkers measurable in blood. NEVs, isolated from peripheral blood using neuronal markers such as L1CAM and ATP1A3, are enriched with AD-related proteins (p-tau181, t-tau, and Aβ_42_/Aβ_40_), miRNAs, and other potential targets, offering a promising platform for early and specific diagnosis of AD. Created with BioRender.com. ATP1A3: ATPase Na^+^/K^+^ transporting subunit alpha 3; Aβ: amyloid-β; AD: Alzheimer’s disease; GFAP: glial fibrillary acidic protein; L1CAM: L1 cell adhesion molecule; miRNAs: microRNAs; NEVs: neuronal-derived extracellular vesicles; NfL: neurofilament light chain; p-tau: phosphorylated tau; t-tau: total tau.

**Key findings of BBMs in AD:** The blood contains both core AD biomarkers (e.g., Aβ_42_/Aβ_40_, p-tau217, p-tau181, and p-tau231) and non-core biomarkers (e.g., neurofilament light chain, glial fibrillary acidic protein, and total tau [t-tau]), offering insight into brain pathology via peripheral samples (**Figure 1**). For instance, plasma Aβ_42_/Aβ_40_, measured by immunoprecipitation coupled with mass spectrometry, can accurately predict amyloid PET status, especially when combined with age and apolipoprotein E (APOE) ε4 status (Schindler et al., 2019). Plasma Aβ_42_/Aβ_40_ can also predict conversion from amyloid PET-negative to amyloid PET-positive, suggesting that plasma Aβ_42_/Aβ_40_ may be used as a cost-effective screening tool for drug trials (Schindler et al., 2019). However, plasma Aβ_42_/Aβ_40_ has shown limited clinical utility due to the small difference between Aβ-positive and Aβ-negative individuals. Peripheral Aβ expression dilutes CNS-specific signals, and assay performance can be affected by pre-analytical factors such as analyte stability and surface adsorption. Additionally, high inter-laboratory variability further compromises the reliability of plasma Aβ measurements (Hunter et al., 2025). To overcome these challenges, alternative strategies are being explored, such as isolating neuronal-derived extracellular vesicles (NEVs) from blood, to enrich CNS-derived Aβ and improve specificity.

Given these limitations of plasma Aβ biomarkers, p-tau has emerged as a more reliable alternative, with clinical studies consistently demonstrating its strong performance in large, real-world cohorts. Among them, plasma p-tau181 correlates strongly with both tau and amyloid PET, as well as CSF p-tau181 levels (Janelidze et al., 2020). It increases with clinical progression and can distinguish AD from cognitively healthy individuals, mild cognitive impairment, and other neurodegenerative diseases (Janelidze et al., 2020). Notably, elevated plasma p-tau181 levels predict future AD development in both cognitively normal individuals and those with mild cognitive impairment. For instance, plasma p-tau181 is increased up to 8 years before death in patients with AD, and when combined with Aβ42 and neurofilament light chain, can anticipate AD onset up to 8 years before clinical symptoms appear (Lantero Rodriguez et al., 2020).

Even more compelling clinical data come from the Swedish BioFINDER study on plasma p-tau217, which has demonstrated superior diagnostic and prognostic value compared to p-tau181 and magnetic resonance imaging-based biomarkers, outperforming them in distinguishing AD from controls and other neurodegenerative diseases (Palmqvist et al., 2020). Plasma p-tau217 also predicts amyloid and tau PET status more accurately than plasma p-tau181 (Palmqvist et al., 2020). In fact, the 2024 Alzheimer’s Association guidelines recommend plasma p-tau217 to diagnose, stage, and prognose AD because it typically outperforms other p-tau assays (i.e., p-tau181, p-tau231) in head-to-head comparisons and has an accuracy comparable to approved CSF assays (Jack et al., 2024). Hyperphosphorylated tau species are preferred because plasma t-tau mainly originates from peripheral sources; thus, CNS-derived increases in t-tau have minimal impact on plasma levels. Nevertheless, NEV-based approaches may offer new opportunities for detecting CNS-derived t-tau more accurately. A summary of the main findings is presented in **Table 1**.

**Table 1:** Highlights on key findings of blood-based biomarkers in Alzheimer’s disease

Biomarker/Approach	Key findings
Aβ_42_/Aβ_40_	Accurately predicts amyloid PET status. Clinical utility is limited due to peripheral Aβ expression and assay variability.
p-tau181	Strongly correlates with amyloid and tau PET, and CSF p-tau181. When combined with Aβ_42_ and neurofilament light chain, predicts Alzheimer’s disease onset years before symptoms.
p-tau217	Shows the highest diagnostic accuracy; recommended by 2024 Alzheimer’s Association guidelines.
t-tau	Limited by peripheral origin, but detection in NEVs may improve specificity.
NEVs	A promising approach to enhance detection of CNS-derived non-core biomarkers.
Affinity-based proteomics	Enables high multiplexing and attomolar sensitivity.

Aβ: Amyloid–beta; CNS: central nervous system; CSF: cerebrospinal fluid; NEVs: neuronal-derived extracellular vesicles; PET: positron emission tomography; t-tau: total tau.

**NEVs as promising BBMs for AD diagnosis:** Extracellular vesicles (EVs) are nanosized, membrane-bound particles released by cells that play a fundamental role in physiological and pathological processes. They are involved in intercellular communication and molecular transport, carrying bioactive molecules such as proteins, lipids, microRNAs, and nucleic acids. These biomolecules reflect the molecular composition of their cell of origin and can provide insights into cellular conditions, making NEVs valuable candidates for AD biomarker discovery (Rocha et al., 2024). Growing evidence suggests that NEV cargo includes core pathological proteins, such as Aβ_42_ and p-tau181, along with non-core AD biomarkers, such as t-tau, which becomes more specific to the CNS when measured in NEVs (Rocha et al., 2024; Hunter et al., 2025; **Figure 1**). NEVs isolated from the blood of AD patients have also shown alterations in markers of insulin signaling, synaptic protein levels, and microRNA expression (Rocha et al., 2024). Importantly, NEVs can cross the blood-brain barrier and accumulating in the bloodstream, allowing access to CNS-specific biomarkers through minimally invasive procedures (Hunter et al., 2025).

A major challenge in using NEVs as biomarkers is their isolation from complex biofluids such as plasma. Various methods are employed for EV isolation, differing in purity, yield, and practicality. Ultracentrifugation has long been considered the gold standard, offering high purity but requiring specialized equipment, large sample volumes, and extensive processing time. Alternative techniques such as ultrafiltration and size-exclusion chromatography provide simpler workflows but often yield lower recovery. Polymer-based precipitation is a simple, high-yield method using commercial kits like ExoQuick ULTRA, but it lacks purity due to co-precipitation of blood proteins and polymer-related interference. Typically, combining multiple isolation techniques can improve EV purity and yield (Hunter et al., 2025). Since standard isolation techniques cannot distinguish NEVs from peripheral EVs, immunoaffinity capture is a common strategy. The L1 cell adhesion molecule is the most frequent target due to its high neuronal expression. However, concerns over the specificity of L1 cell adhesion molecule have driven the search for alternative markers, such as ATP1A3, which demonstrates stronger neuronal enrichment but is still not exclusively neuronal (Rocha et al., 2024). Low recovery remains a significant challenge in EV immunoprecipitation techniques, partly due to the low abundance of NEVs in plasma. Detecting protein targets in isolated NEVs is also difficult, even with ultrasensitive methods. To address this, researchers have turned to single-event techniques, such as nanoparticle tracking analysis and flow cytometry, though these approaches are often semi-quantitative and not yet suitable for clinical diagnostics. More recently, affinity-based proteomic methods, such as Olink and NULISA, have shown promise by providing attomolar sensitivity and high multiplexing capabilities. These technologies have significant potential to enhance the study of blood NEVs as sources of AD-related biomarkers (Rocha et al., 2024; Hunter et al., 2025). Nevertheless, the field still lacks standardized NEV isolation protocols, which are critical for ensuring reliability and reproducibility.

**Current challenges in clinical implementation:** Although BBMs hold strong potential for clinical applications, including early screening, disease staging, therapeutic monitoring, and differential diagnosis of AD, their use remains largely restricted to clinical trials and specialized centers. Broader adoption is currently limited by assay variability, lack of standardized protocols, and the influence of individual-level biological factors. Immunoassays are vulnerable to both pre-analytical and analytical conditions, which are a large source of inter- and intra-assay variability. Standardization of BBM measurements across clinical settings is crucial for the implementation of BBMs. Equally important is the establishment of cutoff concentrations and reference ranges for all BBMs, which are essential to ensure diagnostic accuracy and facilitate translatability (Jack et al., 2024). Beyond assay-related challenges, individual-level factors can influence BBM concentrations and their interpretation. Variables such as age, sex, genotype, and comorbidities, including chronic kidney disease, previous stroke, diabetes, dyslipidemia, and high body mass index, can significantly influence biomarker levels and their interpretation (O’Bryant et al., 2023). Additionally, population diversity, socioeconomic disparities, and lifestyle factors may affect BBM performance, yet studies on BBMs and their influences remain focused on high-income countries (O’Bryant et al., 2023). To address this gap, our group recently validated the performance of BBMs in a Brazilian dementia cohort (Santos et al., 2025). Beyond biological validation, economic implications are critical for the broad clinical adoption of BBM detection technologies, particularly in developing regions. Although BBMs offer a less invasive and more accessible alternative to CSF and PET-based diagnostics, many of the technologies involved, such as SiMoA and immunoprecipitation coupled with mass spectrometry, do remain costly and require specialized equipment and technical expertise, which may limit widespread implementation. Overcoming these challenges requires establishing precise diagnostic thresholds, validating biomarkers across diverse populations, and establishing local cut-off values to enhance clinical applicability and accessibility (Jack et al., 2024).

**Conclusion:** Blood-based biomarkers represent a transformative step toward accessible, cost-effective, and accurate detection and monitoring of AD, offering a viable alternative to traditional CSF and neuroimaging methods. Among them, NEVs provide a promising approach for capturing CNS-specific biomarkers, potentially improving diagnostic accuracy. However, challenges such as standardization, population variability, and the influence of comorbidities remain. Advancing BBMs implementation will require rigorous validation, analytical standardization, refined diagnostic thresholds, and equitable access to testing to ensure their successful integration into clinical practice and ultimately enhance early detection and disease management.
